# Does cervical lordosis change after spinal manipulation for non-specific neck pain? A prospective cohort study

**DOI:** 10.1186/s12998-015-0078-3

**Published:** 2015-12-07

**Authors:** Michael Shilton, Jonathan Branney, Bas Penning de Vries, Alan C. Breen

**Affiliations:** Anglo-European College of Chiropractic, 13-15 Parkwood Road, Bournemouth, BH5 2DF UK; Faculty of Health and Social Sciences, Bournemouth University, Bournemouth House, Bournemouth, BH1 3LH UK; Institute of Musculoskeletal Research and Clinical Implementation, Anglo-European College of Chiropractic, 13-15 Parkwood Road, Bournemouth, BH5 2DF UK

**Keywords:** Neck pain, Spinal manipulation, Cervical lordosis, Sagittal alignment

## Abstract

**Background:**

The association between cervical lordosis (sagittal alignment) and neck pain is controversial. Further, it is unclear whether spinal manipulative therapy can change cervical lordosis. This study aimed to determine whether cervical lordosis changes after a course of spinal manipulation for non-specific neck pain.

**Methods:**

Posterior tangents of C2 and C6 were drawn on the lateral cervical fluoroscopic images of 29 patients with subacute/chronic non-specific neck pain and 30 healthy volunteers matched for age and gender, recruited August 2011 to April 2013. The resultant angle was measured using ‘Image J’ digital geometric software. The intra-observer repeatability (measurement error and reliability) and intra-subject repeatability (minimum detectable change (MDC) over 4 weeks) were determined in healthy volunteers. A comparison of cervical lordosis was made between patients and healthy volunteers at baseline. Change in lordosis between baseline and 4-week follow-up was determined in patients receiving spinal manipulation.

**Results:**

Intra-observer measurement error for cervical lordosis was acceptable (SEM 3.6°) and reliability was substantial ICC 0.98, 95 % CI 0.962–0991). The intra-subject MDC however, was large (13.5°). There was no significant difference between lordotic angles in patients and healthy volunteers (*p* = 0.16). The mean cervical lordotic increase over 4 weeks in patients was 2.1° (9.2) which was not significant (*p* = 0.12).

**Conclusions:**

This study found no difference in cervical lordosis (sagittal alignment) between patients with mild non-specific neck pain and matched healthy volunteers. Furthermore, there was no significant change in cervical lordosis in patients after 4 weeks of cervical spinal manipulation.

## Background

Neck pain is a common complaint that will affect three quarters of people at some point in their lives [1]. It is one of the most commonly reported reasons for ambulatory health care visits with 12 month prevalence rates ranging from 30 to 50 % [2]. At the societal level, neck pain significantly impacts economically in terms of work absenteeism and health care expenditure [3–5].

In general, despite technological advancements, an accurate diagnosis of neck pain remains elusive [6], but it has been proposed that the amount of lordosis (sagittal alignment) in the cervical spine is important for treatment and prognosis [7, 8]. However, the importance of cervical lordosis in relation to neck pain is controversial and has yet to be substantiated by high quality prospective research.

It has been suggested that lordosis can change following trauma or due to disc degeneration [9] and reduced cervical lordosis has been associated with neck pain in acute and chronic neck pain patients [7, 10–12]. However, one study used retrospective data from radiographs ranging from 1988 to 2003 [12], giving rise to concerns about measurement standardisation. Given the time frame, it seems reasonable to suggest that positioning may not have been standardised across time. In addition, others [13–18] found no association between lordosis and neck pain, and in a literature review Gay [16] concluded that the curve of the cervical spine had little prognostic significance. Further, a more recent systematic review concluded that an association between cervical lordosis and spinal pain was not supported by the epidemiological evidence, albeit much of the research reviewed was found to be of low methodological quality [19].

Harrison et al. [7, 11] reported increases in cervical lordosis after treatment (consisting of spinal manipulative therapy (SMT) and cervical traction) in 30 neck pain patients and found this to be consistent with a reduction in pain. However, the authors [7] conceded that their study design fell short of allowing them to suggest that one has caused the other. In addition, if a systematic change in lordosis after treatment is found, this change cannot be attributed to the treatment intervention if there is a lack of (i) a control group with which to compare differences in change or (ii) an estimate of measurement error.

Closer inspection reveals further design problems with these studies [7, 11]. Although they incorporated a standardised radiographic positioning protocol consisting of obtaining two flexion and extension positions reached with eyes closed, this may involve a considerable re-positioning error due to patients not being re-positioned in exactly the same way as for the first measurement. Furthermore, one study [11] involved only patients with a reduced lordosis at baseline and in the other [7] subjects were excluded if they had a cervical kyphosis, either segmentally or throughout the neck. This calls into question the generalisability of the findings.

According to Cooperstein and Gleberzen [20], there is a paucity of evidence investigating the ability of SMT to alter the shape of spinal curves and to our knowledge, no one has established a mimimum detectable change (MDC) to allow one to distinguish real changes from natural variation. Although the Cobb angle analysis has been the method of choice for measurement of overall lordosis and kyphosis of the sagittal spinal curves on lateral radiographs, it has been claimed that the posterior tangent method is superior in terms of measurement error (standard error of measurement) and face validity by avoiding over or under-estimation of lordosis [7, 11, 21].

This present study aimed to explore the effects of cervical manipulation on lordosis as measured using the posterior tangent method.

The study objectives were:

1. To determine the intra-observer and intra-subject repeatability (measurement error and reliability) for cervical lordosis measurement in healthy volunteers
2. To determine whether cervical lordosis changes (change equal to or larger than the MDC calculated from untreated healthy volunteers) after a course of spinal manipulation for non-specific neck pain.

## Methods

### Study design

**Fig. 1:**
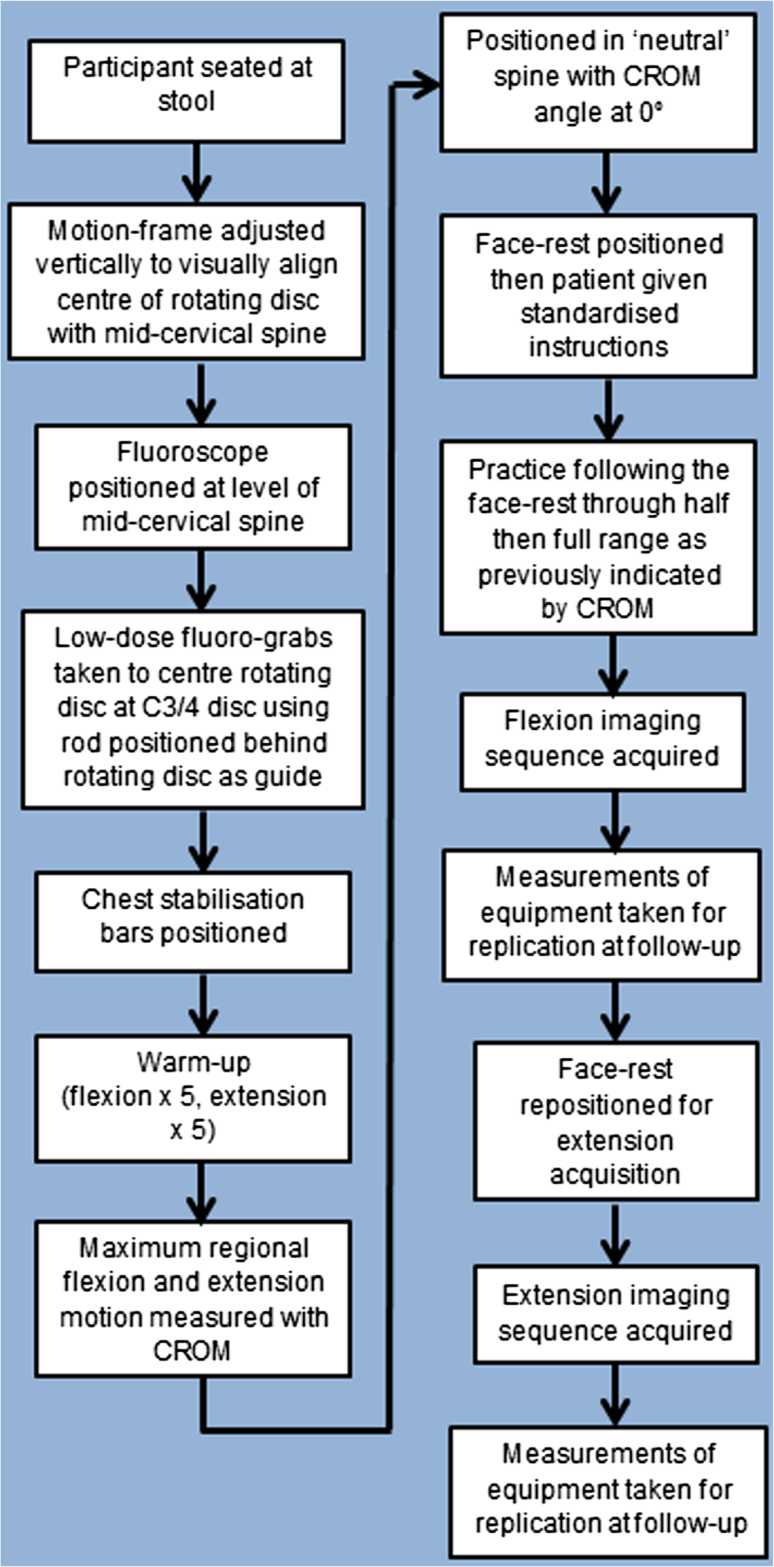
Fluoroscopic image acquisition protocol

The data for this study were collected as part of a prospective cohort study [22] (the ‘parent study’) investigating the effect of spinal manipulation on inter-vertebral motion. In that study, fluoroscopic imaging sequences of cervical flexion/extension were recorded at baseline and 4-week follow-up in neck pain patients receiving SMT and healthy volunteers not receiving any treatment using a standardised positioning protocol (Fig. 1). From those sequences, initial static neutral images were extracted as Audio Video Interleaved (AVI) files from which to measure cervical lordosis in this present study.

**Fig. 2:**
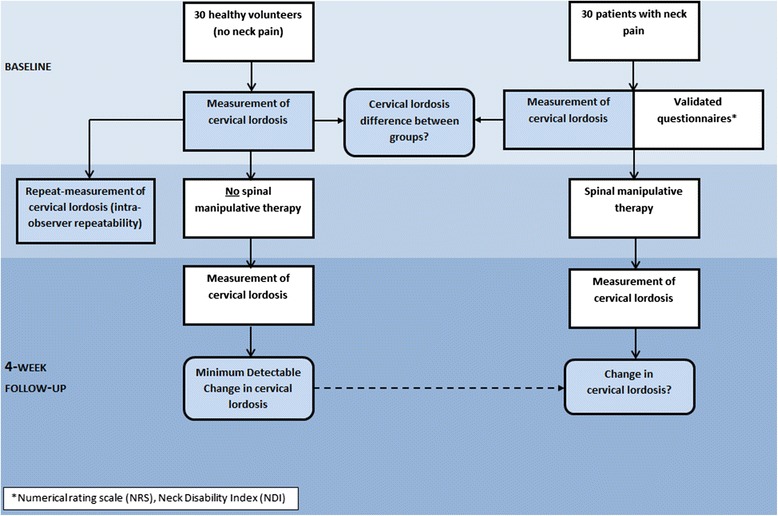
Study flow diagram

The sample size of 30 in each group was a realistic recruitment target given time and resource constraints and would allow adequate opportunity for normal distributions of interval data if present [23]. The sample provided a 90 % power to detect a 6° (SD 10) change in cervical lordosis in patients at the 95 % level of significance, hence the possibility of detecting changes far smaller than those previously reported in response to manual treatment in the literature [7]. Figure 2 provides an overview of the study design.

**Fig. 3:**
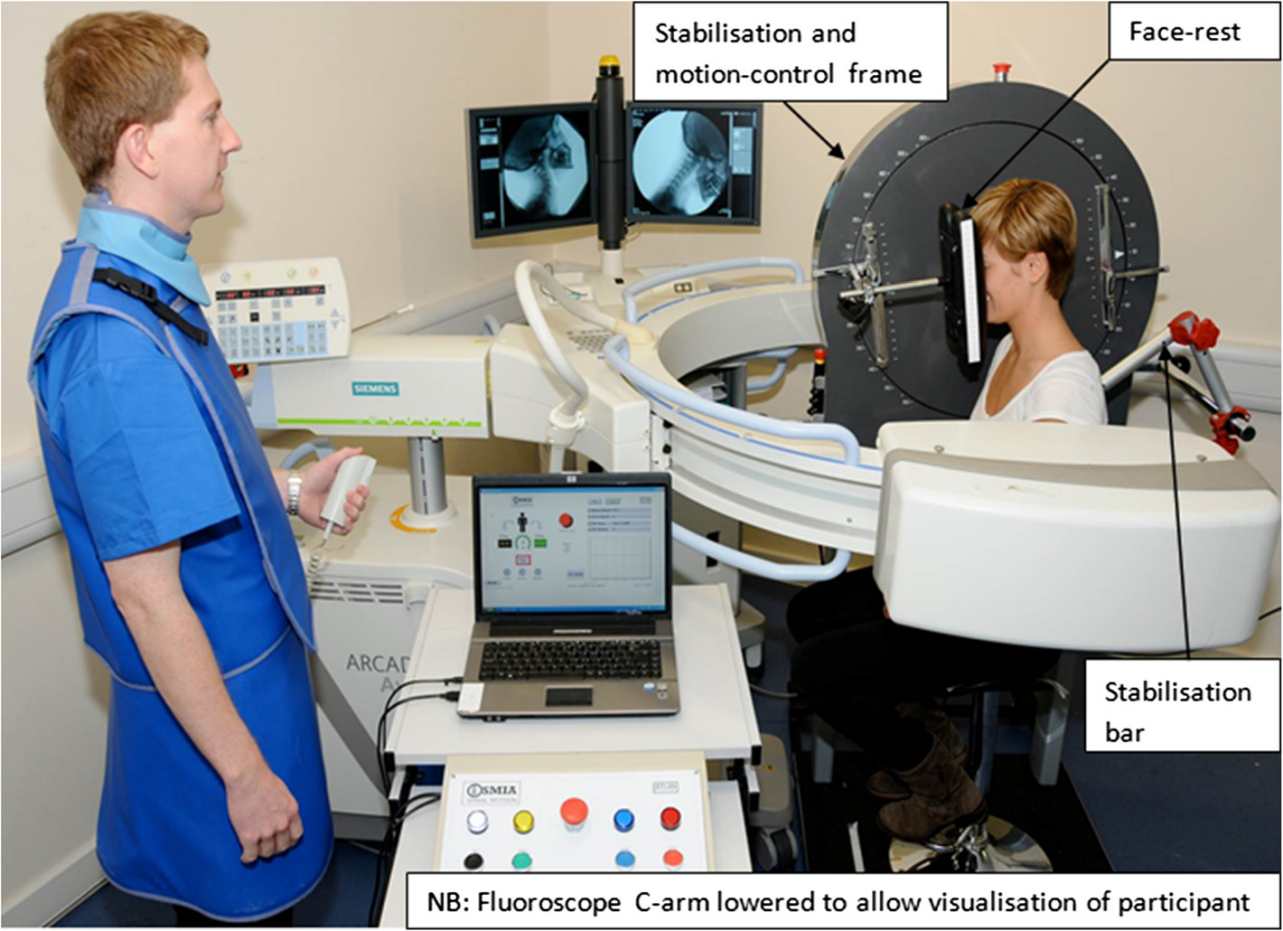
Image acquisition set-up

An intra-observer repeatability study was undertaken to test the repeatability (measurement error and reliability) of the measurement instrument in healthy volunteers (*n* = 30) [24]. The cervical lordoses of non-specific neck pain patients were compared at baseline with healthy volunteers and a baseline to follow-up comparison in healthy volunteers was used to calculate the MDC. Changes in cervical lordosis at follow-up in patients were then identified with respect to the MDC. The acquisition set up of the parent study is shown in Fig. 3.

**Fig. 4:**
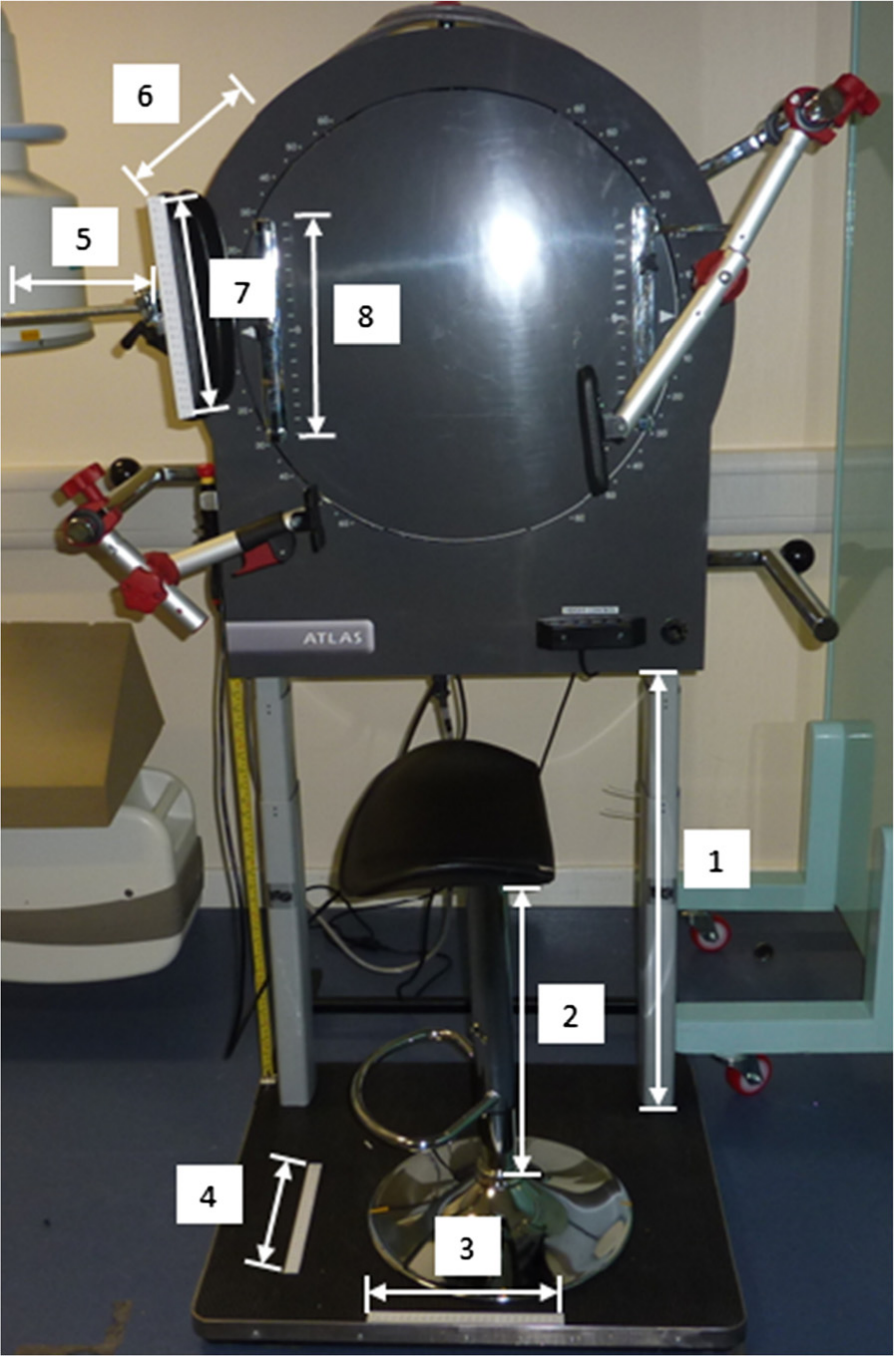
Stabilisation and motion frame with aspects that are measured indicated. Key to Fig. 4: 1. Height of motion frame, 2. Height of stool, 3. Position of stool base, 4. Position of stool base, 5. Horizontal distance of face-rest, 6. Distance from motion-frame to face-rest, 7. Position of participant’s face on face-rest 8. Height of face-rest

Measurements of the positioning apparatus at baseline were taken and recorded (Figs. 3 and 4) so that the configuration could be faithfully replicated at 4-week follow-up.

### Participants

All participants were recruited from August 2011 to April 2013. Data were collected from 30 patients (21 female) attending the Anglo-European College of Chiropractic (AECC) out-patient clinic with a new episode of non-specific neck pain of at least 2 weeks’ duration and 30 pain-free healthy volunteers age and gender-matched with the patients and recruited from staff and students of AECC and the Faculty of Health and Social Sciences (formerly the School of Health & Social Care), Bournemouth University. One patient’s imaging sequence was not available due to a technical error which reduced the patient sample to 29.

The inclusion criteria for patients were: non-specific neck pain (reproducible by neck movement/provocation tests), of at least two weeks' duration, a self-reported pain rating of 3 or more on a 11 point numerical rating scale (NRS) and no suspected pathology.

The inclusion criteria for the healthy volunteers were that they should not have any current neck pain, dizziness or vertigo or any neck pain that limited activity for more than 24 h in the last 12 months.

### Image measurement

**Fig. 5:**
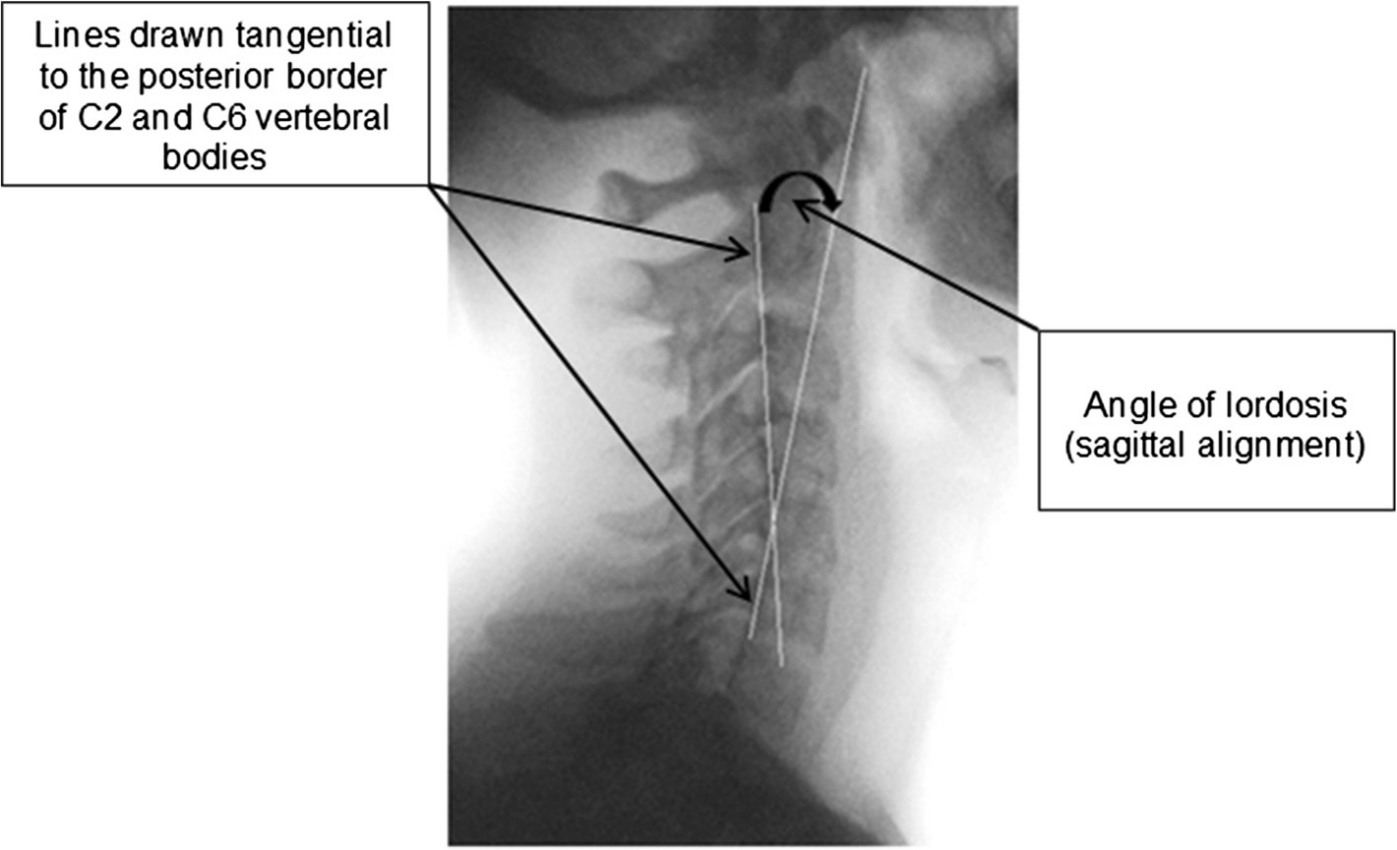
Posterior tangent method of measuring cervical sagittal alignment

For this study, the method of Gore was used for image measurement to be consistent with other studies [7, 8, 11, 14] for comparison and because Harrison et al. [21] found it to be superior to the Cobb method in terms of the measurement error (SEM). This method involves measuring the angle between lines drawn parallel to the posterior surface of the vertebral bodies of C2 and C7 (Fig. 5).

The image measurement was facilitated by importing the fluoroscopic images into ‘Image J’ digital geometric software (available from: http://imagej.nih.gov/ij/ [Accessed June 2013]). As C7 was not visualised in six of the patients and two of the healthy volunteers the vertebral bodies chosen for this measurement throughout the study were C2 and C6. The image used was reduced to 75 % of the original size before marking. Using the program’s drawing tool, a line was drawn posteriorly to the vertebral bodies of C2 and C6 and the protractor tool was then used to measure the angle between them. Kyphotic and lordotic angles were recorded as negative and positive values, respectively.

### Interventions

The intervention involved SMT of the cervical region twice per week for 4 weeks. Manipulation was a high velocity low amplitude thrust (HVLA) using diversified techniques [25] as clinically indicated (based on patient history and exam findings including segmental pain/restriction as identified by static and motion palpation) and delivered by a chiropractor of at least 5 years’ clinical experience. Patients received a mean of 1.3 cervical manipulations per visit (SD 0.4) and 10.7 over the course of the study (SD 3.5) [22]. Final year chiropractic interns also administered trigger point therapy and light massage (both received at least once by 27 patients) to the neck as clinically indicated. Seven patients reported using hot or cold packs during the study period, and 18 used over the counter pain-relieving medication [22]. The outcome measure for this present study was the angle of cervical lordosis.

### Data analysis

Analysis was carried out using IBM SPSS Statistics (V210 and Stats Direct (V2.7.7). Baseline and follow-up lordoses for both patients and healthy volunteers were assessed for normality using the Shapiro-Wilk test. Provided the data were normally distributed, an unpaired two-tailed *t*-test was used to evaluate whether there was a statistically significant difference (significance level α = 0.05) in mean cervical lordosis at baseline between patients and healthy volunteers. Baseline to follow-up comparisons in patients were performed using paired two-tailed *t*-tests (significance level α = 0.05).

Repeatability encompasses measurement error (agreement) and reliability [24]. Measurement error was quantified by the SEM and repeatability coefficients were calculated to represent the MDC [26]. The SEM and MDC in healthy volunteers were calculated using the following formulae:

$$ \begin{array}{l}{\mathrm{S}\mathrm{EM}}_{\mathrm{agreement}}=\sqrt{\mathrm{MSW}}={\mathrm{S}}_{\mathrm{w}}\\ {}\mathrm{M}\mathrm{D}\mathrm{C}={\mathrm{S}}_{\mathrm{w}}\cdotp \kern0.5em \sqrt{2}\cdotp \kern0.5em 1.96\end{array} $$

Here, s_w_ and MSW denote the within-subject standard deviation and within-subjects mean square, respectively. For the intra-*observer* repeatability study, one observer repeated two measurements of cervical lordosis per healthy volunteer from one fluoroscopic image, at least 24 h apart. For the intra-*subject* repeatability study, calculations were based on baseline and follow-up lordosis measurements from each healthy volunteer as obtained by one observer.

Intraclass correlation coefficients (ICC) were used to quantify intra-observer reliability [24]. Generally ICC_agreement (A)_ is the better option over ICC_consistency (C)_ as the first is sensitive to proportional and fixed bias while the later only to proportional bias [27]. Since measurements per subject could potentially differ in a systematic manner, a 2-way analysis of variance (ANOVA) was used to estimate the various components of the ICC parameters. The type of ICC calculated was ICC (3A,1) single measures as each target or object of measurement is rated by each of the same *k* observers, where *k* = 1, and it was assumed that this was the only observer of interest [28, 29]. Using SPSS, ICCs (3A, 1) and 95 % confidence intervals (CI) were obtained.

## Results

Baseline and follow-up measurements of lordoses for both patients and healthy volunteers were normally distributed.

### Participant baseline characteristics

Table 1 shows the baseline characteristics of the healthy volunteers and patients. There were no statistically significant differences between the groups in terms of their gender, age or cervical lordosis, although patients tended to have greater lordotic curves.

**Table 1:** Baseline characteristics of participants

	Patients	Healthy volunteers	Significance (p)
N	29	30	
Female	21	21	
Age, years	39.6 (12.8)	40.5 (12.7)	0.72^*^
NRS score/10	5.1 (1.4)		
NDI score/50	12.7 (6.6)		
Cervical lordosis, degrees	9.5 (13.5)	4.4 (14.0)	0.16^*^

Mean (SD) unless otherwise stated; *NRS* 11-point numerical rating scale; *NDI* neck disability index

^*^ *p*-values for unpaired two-tailed t-tests

### Repeatability (measurement error and reliability) of cervical lordosis measurement

Table 2 shows the intra-observer and intra-subject repeatability in healthy volunteers.

**Table 2:** Intra-observer and intra-subject repeatability in healthy volunteers

	Intra-observer repeatability	Intra-subject repeatability
SEM_agreement_	3.6°	4.9°
MDC	9.9°	13.5°
ICC (3A,1), (95 % CI)	0.98 (0.962–0991)	0.87 (0.743–0.936)

*SEM* standard error of measurement; *MDC* minimum detectable change; *ICC (3A, 1)* intra-class correlation coefficient two-way single measures mixed effects model (agreement)

An intra-observer MDC of 9.9° indicates that two measurements performed by one observer within 24 h and using one radiograph are expected to differ by no more than 9.9° in 95 % of subjects [24]. Similarly, an intra-subject MDC of 13.5° indicates that over a 4-week period subjects’ lordosis measurements are expected to change no more than 13.5°. Only changes greater than 13.5° can, at least in part, be confidently associated with a factor (such as treatment) to which the healthy volunteers have not been exposed.

### Changes to cervical lordosis in patients

Patients’ lordoses increased, on average, from +9.5° (SD 13.5°, 95 % CI 4.6°–14.5°) to +11.6° (SD 11.8°, 95 % CI 7.3°–15.9°). These changes were not statistically significant (*p* > 0.05). The change in cervical lordosis was highly variable (range = 0.1–24.9°). In only 14 % (4/29) of patients was cervical lordosis increased by at least the MDC.

## Discussion

Many researchers have suggested that a loss of cervical lordosis, as measured using plain-film radiographs, might be a cause of neck pain [7, 8, 10–12]. This has led some practitioners to place emphasis on the restoration of the lordotic curve as an important outcome measure for their treatment [8, 11, 30]. However, other researchers have suggested that a lack of lordosis is a normal variant and therefore not a cause of symptoms for neck pain [14–18].

In order to determine whether cervical lordosis changes because of treatment, a measurement tool of high repeatability is required to detect small differences. No studies were discovered in this review of the literature that found patients with neck pain to have a different cervical lordosis from asymptomatic subjects using a methodology that does not involve exclusion based on pre-existing cervical spine alignment or with highly standardised positioning.

The present investigation used images in which the cervical lordoses of clinically presenting neck pain patients matched with healthy volunteers were measured under highly standardised positioning at baseline and 4 week follow-up. In this way it was possible to more confidently investigate the association between cervical lordosis and pain and to test the repeatability of measuring cervical lordosis.

### Intra-observer repeatability

The ICC (3A,1) of 0.981 (0.962–0991) indicates substantial reliability [31]. However, the intra-observer study demonstrated only modest levels of agreement with an SEM of 3.6°. This is higher than that reported by Gwinn et al. [32] and three times higher than that reported by Jackson et al. [33]. However, Jackson et al. [33] did not report which type of SEM was calculated (SEM_consistency_ or SEM_agreement_). Further reasons for their lower SEM could be having better image quality (plain film as opposed to fluoroscopic images) and/or more experienced observers.

### Cervical lordosis in non-specific neck pain patients versus healthy volunteers

There was a non-statistically significant baseline difference (mean = 5.1°) in lordosis between patients and healthy volunteers, with the patients having the greater lordosis. However, this difference was not detectable in the current study. Based on a standard deviation of 14° (see Table 1, healthy volunteers), a sample size of at least 166 patients and 166 healthy volunteers would be required in order to detect a difference of 5° in lordosis with a statistical power of 90 % and significance level of 0.05. Thus, the non-significance for the difference may have been due to a type 2 error. Furthermore, while significant differences might be detected at the group-level with a sufficiently large sample size the large individual variability in cervical lordotic angles (−18–32° in patients and −22–36° in healthy volunteers) means that this is not a feasible technique for the evaluation of individual patients.

### Cervical lordosis of patients at baseline and 4 week follow-up

The results from this study showed a mean increase in cervical lordosis in the patient group of 2.1° (SD 9.2°). This was not statistically significant and well below the natural variation in the healthy volunteers (MDC 13.5°). To attain a statistical power of 90 % with a 0.05 significance level, a sample size of at least 437 patients would be required to detect a mean difference of 2.1° in lordosis between baseline and follow-up, however this difference is not likely to be clinically meaningful. Two studies in the literature have attempted to measure change in cervical lordosis and have reported mean increases above 13.5°.

Harrison et al. found a 14.2° [11] and 17.9° [7] change in neck pain patients. In both of these studies the authors reported an increase in cervical lordosis coupled with a reduction in pain, but did not report the MDC or present a power calculation. The treatment groups received SMT for three weeks [11] and four weeks [7] and then a further traction period of nine weeks [11] and 14 weeks [7]. As the results from our study suggest that there is no association between cervical lordosis and pain it appears initially at odds with the Harrison studies. However, any changes in cervical lordosis that were achieved in those studies were perhaps due to traction rather than SMT [7] but that remains unknown. In the absence of randomisation or a control group there is also the possibility that these changes were due to natural variation (independent of treatment).

### Strengths, limitations and suggestions for further research

A strength of this investigation lies in its use of prospective data of clinically presenting patients of all cervical sagittal alignments to be radiographically imaged under highly standardised conditions. In addition, the present study measured and reported both the measurement error and reliability of the method.

The MDC that was calculated from the healthy volunteers in this study, which provides information on the natural fluctuation of cervical lordosis over time, does not appear to have been previously reported. This suggests that small intervention effects on cervical lordosis will be difficult to detect. An MDC derived from a symptomatic cohort rather than asymptomatic subjects would give greater confidence in determining whether a change in treated symptomatic subjects could be attributed in part to the treatment, although this would present the ethical and practical challenges of recruiting patients who would consent to receiving no manual treatment.

While no significant difference in cervical lordosis was found between patients and healthy volunteers that does not preclude such a difference being detected in a study with a sufficiently large sample size. A further limitation of this study is that its design does not allow us to establish a causal relationship between cervical lordosis and pain, nor did it address other clinical outcomes. In addition, because six of the patients and two of the healthy volunteers had images where C7 could not be visualised, the study used C2-6 throughout, unlike previous studies [7]. However, this was thought not to be critical as the angle difference between C6 and C7 is considered to be very small [33].

Finally, it is noted that the width of the line drawn and decisions regarding accommodating osteophytes require interpretation and practice to develop consistency. This may be a further important source of variability in measurement.

## Conclusions

This study found no difference in cervical lordosis (sagittal alignment) between patients with mild non-specific neck pain and matched healthy volunteers. Furthermore, there was no significant change in cervical lordosis in patients after 4 weeks of cervical SMT.

## Consent

Written informed consent was obtained from the participant in Fig. 3 for the publication of this report and any accompanying images.
